# Structural Characterization of the *Acer ukurunduense* Chloroplast Genome Relative to Related Species in the *Acer* Genus

**DOI:** 10.3389/fgene.2022.849182

**Published:** 2022-07-14

**Authors:** Weichao Ren, Chi Liu, Song Yan, Zhehui Jiang, Tianhao Wang, Zhen Wang, Meiqi Zhang, Meiqi Liu, Jiaying Sun, Jinhui Gao, Wei Ma

**Affiliations:** ^1^ School of Pharmacy, Heilongjiang University of Chinese Medicine, Harbin, China; ^2^ Faculty of Electrical Engineering and Information Technology, Technical University of Chemnitz, Chemnitz, Germany; ^3^ School of Forestry, Northeast Forestry University, Harbin, China; ^4^ Yichun Branch of Heilongjiang Academy of Forestry, Yichun, China; ^5^ Jiangsu Kanion Pharmaceutical Co. Ltd., Lianyungang, China; ^6^ State Key Laboratory of New-tech for Chinese Medicine Pharmaceutical Process, Lianyungang, China; ^7^ Key Laboratory of Basic and Application Research of Beiyao (Heilongjiang University of Chinese Medicine), Ministry of Education, Harbin, China

**Keywords:** *Acer ukurunduense*, Aceraceae, chloroplast genome, phylogenetic analysis, species identification

## Abstract

*Acer ukurunduense* refers to a deciduous tree distributed in Northeast Asia and is a widely used landscaping tree species. Although several studies have been conducted on the species’ ecological and economic significance, limited information is available on its phylo-genomics. Our study newly constitutes the complete chloroplast genome of *A. ukurunduense* into a 156,645-bp circular DNA, which displayed a typical quadripartite structure. In addition, 133 genes were identified, containing 88 protein-coding genes, 37 tRNA genes, and eight rRNA genes. In total, 107 simple sequence repeats and 49 repetitive sequences were observed. Thirty-two codons indicated that biased usages were estimated across 20 protein-coding genes (CDS) in *A. ukurunduense*. Four hotspot regions (*trnK-UUU/rps16*, *ndhF/rpl32*, *rpl32/trnL-UAG*, and *ycf1*) were detected among the five analyzed *Acer* species. Those hotspot regions may be useful molecular markers and contribute to future population genetics studies. The phylogenetic analysis demonstrated that *A. ukurunduense* is most closely associated with the species of Sect. *Palmata*. *A. ukurunduense* and *A. pubipetiolatum* var. *pingpienense* diverged in 22.11 Mya. We selected one of the hypervariable regions (*trnK-UUU/rps16*) to develop a new molecular marker and designed primers and confirmed that the molecular markers could accurately discriminate five *Acer* species through Sanger sequencing. By sequencing the cp genome of *A. ukurunduense* and comparing it with the relative species of *Acer*, we can effectively address the phylogenetic problems of *Acer* at the species level and provide insights into future research on population genetics and genetic diversity.

## Introduction

The *Acer* L. genus comprises approximately 200 species and has a dominant role in the forests of the Northern Hemisphere (Stace and Hind, 1997; Bi et al., 2016; Gao et al., 2020). *Acer* L. species have been used for several thousand years in China, have gained high economic value, and are now among the most important timber trees (Wu et al., 2008; Saeki and Murakami 2009; Dormontt et al., 2020). Flavonoids, tannins, terpenoids, sterols, alkaloids, diarylheptanoids, diarylheptanoids, and others have been isolated from *Acer*, possessing high bioactivity and exerting pharmacological effects (Tang et al., 2012; Ren and Feng, 2018; Yang et al., 2018; Hou et al., 2019; Liang et al., 2019; Piao et al., 2020; Kausar et al., 2021). Additionally, in Chinese folk medicine, *Acer* has also been widely used to treat angina pectoris, coronary artery cirrhosis, and cardiac–cerebral vascular disease*. A. ukurunduense* mainly grows in broadleaf forests at altitudes of 300–1,500 m, with distribution in northeast China, North Korea, and the Russian Far-East. It is an important autumn leaf-viewing tree in northeast China and can be used as a street tree or in a landscape garden for landscaping. Despite its importance, the phylogenetic relationship of the genus remains unclear (Tian et al., 2002).

The whole chloroplast (cp) genome of tobacco was first described in 1986 (Shinozaki et al., 1986; Daniell et al., 2016). The cp genome comprises multi-functional organelles playing a significant role, such as photosynthesis and the carbon cycle (Singh et al., 2009; Schmidt et al., 2020; Zupok et al., 2021). Generally, the cp genome is more conserved than the nuclear genome, a circular molecule of double-stranded DNA. It usually has a quadripartite configuration consisting of a pair of inverted repeat (IR) regions separated by a large single copy (LSC) region and a small single copy (SSC) region (Eid 2009; Wicke et al., 2011; Shahzadi et al., 2020). The cp genome of land plants ranges from 120 to 170 kb in size and gene order and can thus provide plenty of genetic information for investigating evolutionary divergence and the inter-specific relationships of plants (Palmer, 1985; Wolfe et al., 1987; Daniell et al., 2016). Cp information has been extensively applied in analyses of plant genetic diversity, conservation genetics, and molecular marker development (Moore et al., 2010; Li et al., 2016; Gao et al., 2018; Cui et al., 2019).

Studies have indicated that in many forests throughout the Northern Hemisphere, *Acer* is a dominant genus in terms of abundance or total basal area but that it may also function as a foundation genus (Ellison et al., 2019). Qiao et al. (2020) used data to identify a suite of candidate foundation species in Chinese forests. *A. ukurunduense* and *A. barbinerve* were the only two species that met the criteria as candidate foundation species. *Acer* is the foundation species in many terrestrial ecosystems of Northern China. In a previous study, these five *Acer* species (*A. mandshuricum*, *A. tataricum*, *A. pictum*, *A. tegmentosum,* and *A. ukurunduense*) are widely distributed in the Xiaoxing’an Mountains of China and play important roles in different forest types (Hou and Wang, 2013; Wen, 2021). Currently, cp genome data have been obtained for numerous species of *Acer* (Zhao et al., 2018; Fu et al., 2020; Xia et al., 2020; Dong et al., 2021; Xia et al., 2022). However, no cp genome data were reported for *A. ukurunduense*, which is endemic to Northeast China. The evolutionary history and phylogenetic relationship of the genus *Acer* and the family Aceraceae have not been reported.

In the present study, we assembled a complete cp genome sequence for *A. ukurunduense* using the Illumina sequencing platform. The objective was to study the cp genome structure features of *A. ukurunduense*, determine its effectiveness in establishing species phylogeny, assess its structural efficacy in taxon delimitation, and divergence time was estimated based on the cp genome. In addition, we are interested in identifying molecular markers in the cp genomes of five *Acer* species distributed in the Xiaoxing’an Mountains of China. The present study aimed to reveal the phylogenetic position of *A. ukurunduense* and provide insights into *Acer* evolution.

## Materials and Methods

Supplementary Figure S1 provides a technology roadmap to help the reader better understand the study.

### Plant Material and DNA Sequencing

Whole and fresh green leaves of *A. ukurunduense* were collected in Yichun, Heilongjiang Province, China. The voucher herbarium was deposited in the Pharmacy College of the Heilongjiang University of Chinese Medicine under the voucher number YCL20210606001. Total genomic DNA was isolated from leaves (100 mg) according to the modified CTAB method (Porebski et al., 1997). The remaining samples and DNA extraction were stored at −80°C. In addition, we selected the available complete cp genomes of four other *Acer* species from GenBank, including *A. mandshuricum* (NC_049164), *A. tataricum* (MK479223), *A. pictum* (NC_049127), and *A. tegmentosum* (NC_056233).

### Cp Genome Assembly and Annotation

We produced a 150-bp paired-end library and sequenced it on the Illumina sequencing platform (Benagen, Wuhan, China). The clean reads were filtered using the SOAPnuke v1.3.0 pipeline to obtain plastid-like reads. Filter raw data parameters as follows: 1) remove sequences with N content exceeding 10% of the read length base number, 2) the number of low-quality bases exceeds 50% of the number of bases in the sequence, and 3) contain sequences with unremoved joints, and finally obtain high-quality clean data. We assembled the filtered reads based on the GetOrganelle v1.7.5 (Jin et al., 2020) program with published cp genomes as a reference (Xia et al., 2022). Regarding the genome annotation, we annotated the cp genome of *A. ukurunduense* by CPGAVAS2 (http://47.96.249.172:16019/analyzer/annotate) (Shi et al., 2019) and then manually adjusted and annotated genes. The complete cp genome sequence and gene annotation of *A. ukurunduense* were submitted to the public GenBank database (MZ736145). Then, we used Chloroplot (https://irscope.shinyapps.io/Chloroplot/) to generate a circular cp genome map (Zheng et al., 2020).

### Comparative Cp Genome Analyses

The mVISTA program (https://genome.lbl.gov/vista/mvista/submit.shtml) (Frazer et al., 2004), which applied the Shuffle-LAGAN model (Brudno et al., 2003), was used to align, annotate, and visually compare the cp genome of *A. ukurunduense* with the cp genomes of four *Acer* species (*A. mandshuricum*, *A. tataricum*, *A. pictum*, and *A. tegmentosum*). We chose *A. mandshuricum* as the reference.

### IR/SC Boundary Analysis, Codon Usage, and Genetic Divergence Analyses

The sizes and junction sites of the LSC/IRb/SSC/IRa regions of the five *Acer* species were compared using the IRscope online program (https://irscope.shinyapps.io/irapp/) (Amiryousefi et al., 2018). In the genome structural rearrangement analysis, the five species’ cp genomes were aligned using the MAUVE program (Darling et al., 2004). Condon usage, synonymous codon usage, and RSCU were determined using CodonW v1.4.2. Nucleotide divergence of *Acer* species was calculated based on nucleotide diversity values by employing DnaSP v6 (Rozas et al., 2017) software. Before analysis, we excluded gaps and missing data.

### Repeat Sequences and SSR Analysis

Reverse, forward, complement, and palindromic repeats were then identified within the five species’ cp genomes by the REPuter (https://bibiserv.cebitec.uni-bielefeld.de/reputer) (Kurtz et al., 2001) program, with parameters set as follows: 1) the minimal repeat sequence size was 20 bp, 2) the maximum computed repeat was 50, 3) the identity was not less than 90%, and 4) the hamming distance was 3. The SSR loci of *A. ukurunduense* cp genomes were located using MISA (v2.1) software (http://pgrc.ipk-gatersleben.de/misa/) (Beier et al., 2017). The minimum numbers were: eight repeats for mononucleotide types; four repeats for dinucleotide types; four repeats for trinucleotide types; and three repeats for tetranucleotide types, pentanucleotide types, and hexanucleotide types. The shortest size between any two SSR loci was more than 100 bp.

### Phylogenetic Relationship Analyses and Estimation of the Divergence Time

To parse the phylogenetic status of *Acer* species within the Aceraceae family, multiple sequence alignments were generated using the whole cp genome sequences of 105 *Acer* species and downloaded from the NCBI database (Supplementary Table S1), with *Sapindus mukorossi* and *Arabidopsis thaliana* as outgroups. The cp genome sequence alignment analysis of the 107 species was generated using MAFFT (v7.481) (Katoh et al., 2019) with default parameters. The maximum likelihood (ML) tree (with default parameter settings and 1,000 bootstrap repeats) was performed using the IQ-TREE (v2.1.3) (Lanfear et al., 2020) software with the best-fitting model TVM + F + R5. A Bayesian inference (BI) analysis was performed using the Markov Chain Monte Carlo (MCMC) algorithm in Mr. Bayes, using 1,000,000 generations, a sampling frequency of 100, and default values for other parameters. Visualizing and refining the output tree was implemented in MEGA X.

A timetree was inferred using the ReltimeML-option in MEGA X. The reference-node age was obtained by the divergence time of *A. mandshuricum*–*A. fabri* (20.9–32.5 million years ago) and *A. acuminatum*–*A. saccharum* (11.7–31.9 million years ago) (http://www.timetree.org/, accessed on June 8, 2022).

### Validation of Molecular Markers

Variable intergenic regions can be used to develop molecular markers to discriminate among the five species. We selected the *trnK-UUU/rps16* hypervariable region as an amplified fragment. The primer was designed using the Primer 3 input online software (https://primer3.ut.ee/), and the primer is shown in Supplementary Table S2. The PCR reaction system is as follows: 12.5 µl 2×Taq PCR Master Mix, 1 µl for each of the 10 µM forward and reverse primers, 2 µl total DNA, and 8.5 µl ddH_2_O. PCR amplification procedures with the following conditions: denaturation at 94°C for 2 min, followed by 35 cycles of 94°C for 30 s, 58°C for 30 s, 68°C for 1 min, and a final extension at 68°C for 7 min. PCR products (5 µl) were visualized on 1% agarose gels and then subjected to Sanger sequencing via the ABI3730XL Sequencing platform (Comate Bioscience Co., Ltd. Changchun, China).

## Results

### Chloroplast Genome Features of *A*. *ukurunduense*

**FIGURE 1:**
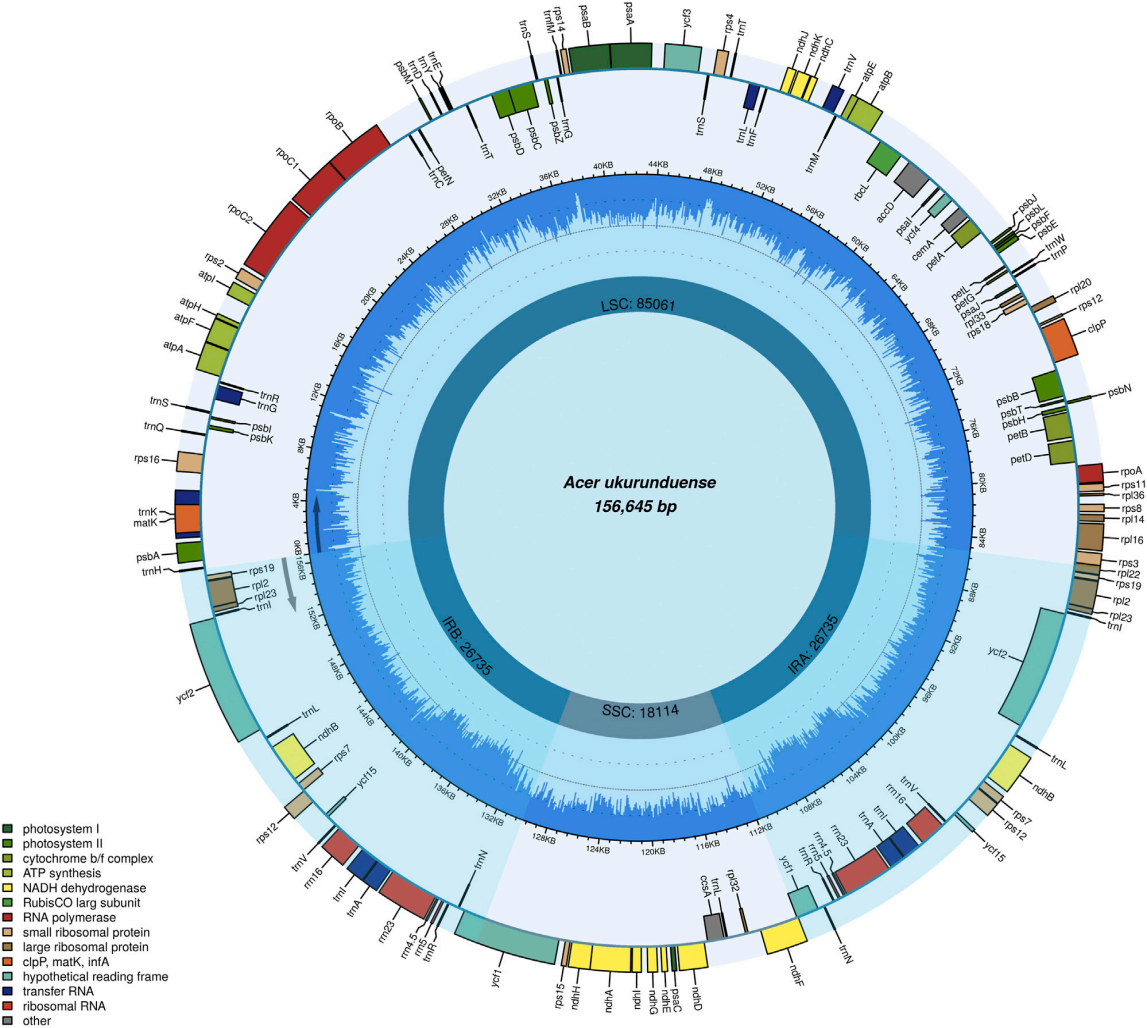
Gene map of the *A. ukurunduense* complete cp genome. Genes drawn within and out of the circle are transcribed in the clockwise and anticlockwise directions, respectively. Genes are color-filled, which represents different functions.

The *A. ukurunduense* cp genome is 156,645 bp, including the LSC region of 85,061 bp, the SSC region of 18,114 bp, and a pair of inverted repeat regions (IRa and IRb) of 53,470 bp (Figure 1). The average GC content ratio is 37.9%, and the average AT content ratio is 62.1%. In terms of gene content and composition, the features of the *A. ukurunduense* cp genome are similar to those of other previously researched *Acer* species. One-hundred thirty three genes were encoded, including 88 code protein genes, eight rRNAs and 37 tRNAs. We can put them into four classes according to their function, including genes for photosynthesis, self-replication, other genes, and unknown (Table 1). We detected a total of 20 genes are repeated in the IR regions, among which four ribosomal RNA genes (*rrn4.5*, *rrn5*, *rrn16S*, and *rrn23*), seven transfer RNA genes (*trnA-UGC*, *trnI-CAU*, *trnI-GAU*, *trnL-CAA*, *trnN-GUU*, *trnR-ACG*, and *trnV-GAC*), and nine PCGs (*ndhB*, *rpl2*, *rpl23*, *rps12*, *rps19*, *rps7*, *ycf1*, *ycf2*, and *ycf15*). Twenty-two genes contain introns. There are two genes (*ycf3* and *clpP*) containing two introns, and the other 20 genes contain one intron (Supplementary Table S3).

**TABLE 1:** Gene list of cp genome in *A. ukurunduense*.

Category	Group of genes	Name of genes
Self replication	rRNA genes	*rrn4.5* ^a^ *, rrn5* ^a^ *, rrn16S* ^a^ *, rrn23* ^a^
	tRNA genes	*trnA-UGC* ^a^ ^,^ ^b^ *, trnC-GCA, trnD-GUC, trnE-UUC, trnF-GAA, trnfM-CAU, trnG-GCC, trnG-UCC* ^b^ *trnH-GUG, trnI-CAU* ^a^ *, trnI-GAU* ^a^ ^,^ ^b^ *, trnK-UUU* ^b^ *, trnL-CAA* ^a^ *, trnL-UAA* ^b^ *, trnL-UAG, trnM-CAU, trnN-GUU* ^a^ *, trnP-UGG, trnQ-UUG, trnR-ACG* ^a^ *, trnR-UCU, trnS-GGA* ^a^ *, trnS-UGA, trnT-GGU, trnT-UGU, trnV-GAC* ^a^ *, trnV-UAC* ^b^ *, trnW-CCA, trnY-GUA*
	Large subunit of ribosome	*rpl14, rpl16* ^b^ *, rpl2* ^a^ ^,^ ^b^ *, rpl20, rpl22, rpl23* ^a^ *, rpl32, rpl33, rpl36*
	DNA dependent RNA polymerase	*rpoA, rpoB, rpoC1* ^b^ *, rpoC2*
	Ribosome	*rps11, rps12* ^a^ *, rps14, rps15, rps16* ^b^ *, rps18, rps19* ^a^ *, rps2, rps3, rps4, rps7* ^a^ *, rps8*
Photosynthesis	ATP synthase	*atpA, atpB, atpE, atpF* ^b^ *, atpH, atpI*
	Photosystem II	*psbA, psbB, psbC, psbD, psbE, psbF, psbI, psbJ, psbK, psbL, psbM, psbN, psbT, psbZ, ycf3* ^c^
	NADH-dehydrogenase	*ndhA* ^b^ *, ndhB* ^a^ ^,^ ^b^ *, ndhC, ndhD, ndhE, ndhF, ndhG, ndhH, ndhI, ndhJ, ndhK*
	cytochrome b/f complex	*petA, petB* ^b^ *, petD* ^b^ *, petG, petL, petN*
	Photosystem I	*psaA, psaB, psaC, psaI, psaJ*
	Rubisco	*rbcL*
Other genes	Acetyl-CoA-carboxylase	*accD*
	c-type cytochrom synthesis gene	*ccsA*
	Envelop membrane protein	*cemA*
	Protease	*clpP* ^c^
	Maturase	*matK*
Unkown function	Conserved open reading frames	*ycf1* ^a^ ^,^ ^b^ *, ycf15* ^a^ *, ycf2* ^a^ *, ycf4*

a Gene with copies.

b Genes with one intron.

c Genes with two introns.

### Codon Usage Analysis

**FIGURE 2:**
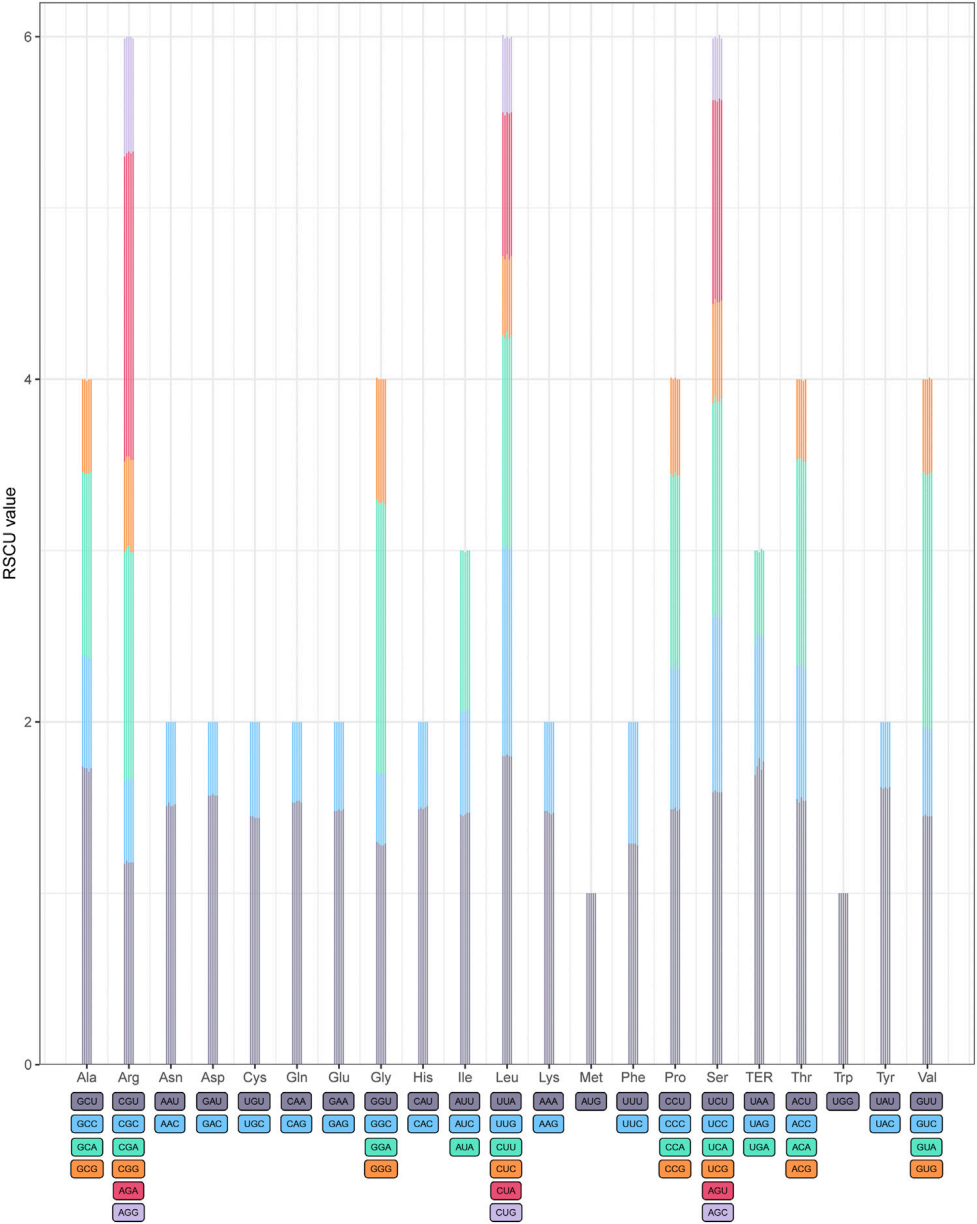
Codon content of twenty amino acids in all protein-coding genes of five *Acer* species cp genomes. The histogram of each amino acid indicated codon usage within *Acer* (from left to right: *A. mandshuricum*, *A. pictum*, *A. tataricum*, *A. tegmentosum*, and *A. ukurunduense*).

The relative synonymous codon usage (RSCU) frequency was estimated in the five *Acer* species (Figure 2). A total of 20 amino acids were detected for protein biosynthesis by tRNA molecules in the cp genomes of the five species, and the relative synonymous codon usage (RSCU) values are listed in Supplementary Table S4. The codon numbers in all combined sequences (CDS) were *A. mandshuricum* (26,677), *A. tataricum* (26,660), *A. pictum* (26,682), *A. tegmentosum* (26,716), and *A. ukurunduense* (26,885), respectively. Among all codons, the most abundant codons encoded were leucine (AUU) (1,091–1,114), and the least abundant codons encoded were L-cysteine (UGC) (82–84). We were surprised to find that the RSCU values of 30 codons and one stop codon (UAA) were greater than 1 after the statistics of the codon RSCU values of five species. For *A. pictum*, there are 27 codons that are end-rich in A or U, and in the other four species, there are 28 codons that are end-rich in A or U, which among all codons whose RSCU value was greater than 1 did not contain a stop codon (UAA). These results indicate that the cp genomes of five *Acer* genus species tend to end with A or U.

### Long-Repeat and Simple Sequence Repeat (SSR) Analysis

**FIGURE 3:**
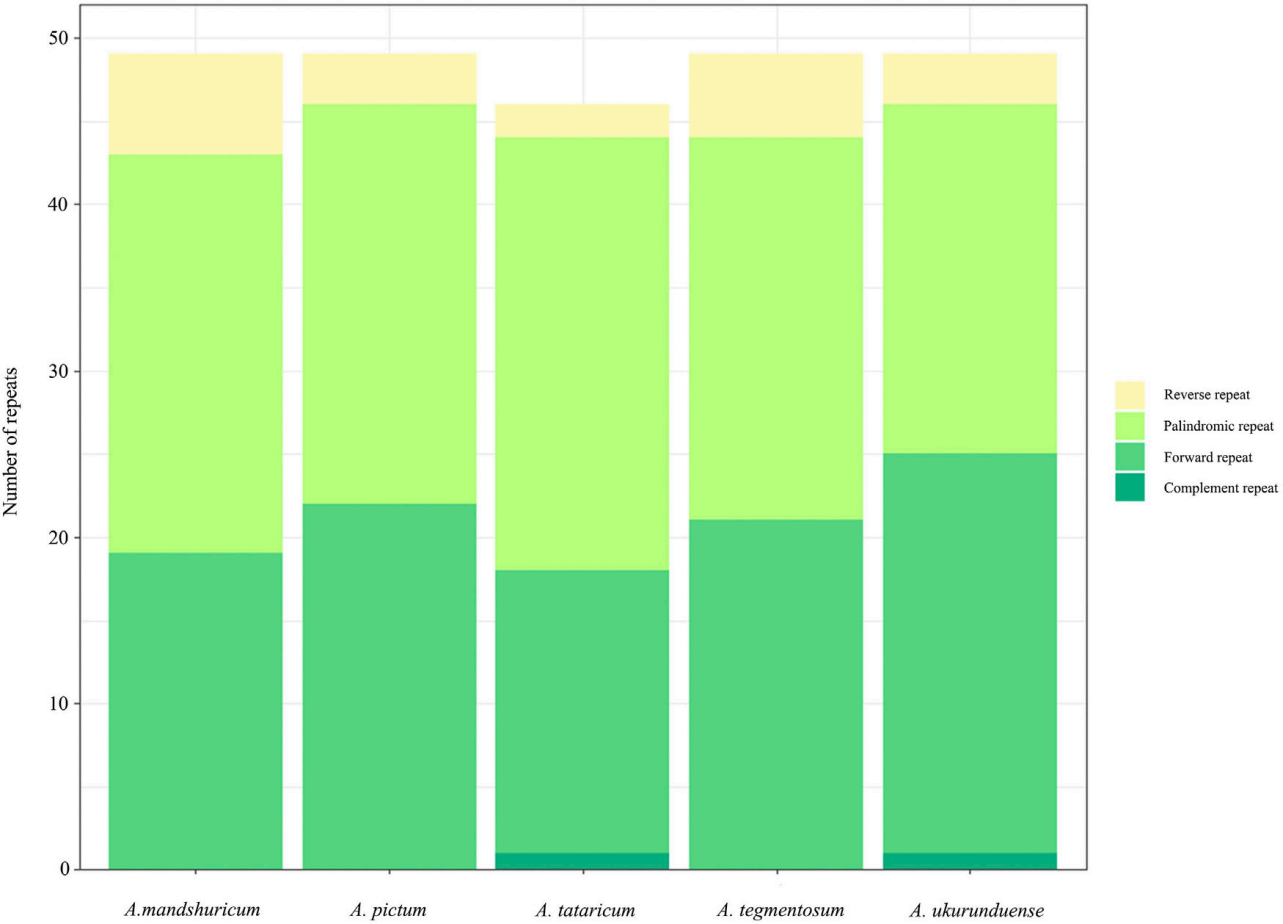
Repeated sequences in five *Acer* cp genomes.

Four species of tandem repeats were presented in this study, including forward, reverse, complement, and palindrome. The detailed number of tandem repeats for each species is illustrated in Figure 3. Expect for *A. tataricum*, the number of tandem repeats was 49. Only *A. tataricum* and *A. ukurunduense* were included in a complement repeat. In addition, the other numbers of three repeats are also different. There were 46 (two reverse, 26 palindromic, 17 forward, and one complement), 49 (six reverse, 24 palindromic, and 19 forward), and 49 (three reverse, 24 palindromic, and 22 forward), 49 (five reverse, 23 palindromic, and 21 forward), 49 (three reverse, 21 palindromic, 24 forward, and one complement) long repeats in *A. tataricum*, *A. mandshuricum*, *A. pictum*, *A. tegmentosum,* and *A. ukurunduense*, respectively (Supplementary Table S5).

**FIGURE 4:**
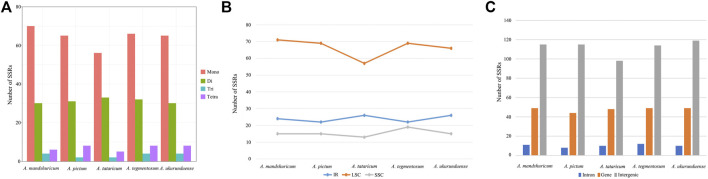
Number of different types of SSRs from five *Acer* species cp genomes. **(A)** Number of different SSR types detected in five cp genomes. **(B)** Number of SSRs in LSC, SSC, and IR regions. **(C)** Number of SSRs in the intergenic regions, genes, and introns.

The analysis of SSRs exhibited four types of SSRs (Table 2). The total number of SSRs was 96 in *A. tataricum*, 109 in *A. mandshuricum*, 106 in *A. pictum*, 110 in *A. tegmentosum*, and 107 in *A. ukurunduense.* The types and content of motifs are different between species. In five species, mono-nucleotide has the largest proportion (58.33%–64.22%), followed by di-nucleotide (27.52%–34.38%), tetra-nucleotide (5.21%–7.55%), and tir-nucleotide (1.89%–3.74%). A mononucleotide accounted for 86.44%–93.84%. The majority of SSRs in all species were A/T mononucleotides. In the cp genome, the LSC region has the most SSRs, ranging from 57 to 71 in the five species analyzed here. However, the number of SSRs in IR regions from 22 to 24 is the lowest, from 13 to 19 in the SSC region, and from 57 to 71 in the LSC region (Figure 4B). The number of SSRs is the largest in the intergenic and the number of SSRs is the lowest in the intron (Figure 4C).

**TABLE 2:** Numbers of simple sequence repeats (SSRs) in five *Acer* cp genomes.

SSR type	Repeat unit	Amount				
		*Acer tataricum*	*Acer mandshuricum*	*Acer pictum*	*Acer tegmentosum*	*Acer ukurunduense*
Mono	A/T	51	63	61	60	59
	C/G	5	7	4	6	6
Di	AC/GT	2	1	2	2	1
	AG/CT	13	13	13	13	13
	AT/AT	18	16	16	17	16
Tri	AAG/CTT	1	1	1	1	1
	AAT/ATT	1	3	1	3	3
Tetra	AAAT/ATTT	-	2	2	1	2
	AACT/AGTT	1	1	1	1	1
	AATC/ATTG	1	-	1	2	1
	AATT/AATT	-	-	1	1	-
	ACAT/ATGT	1	1	1	1	1
	AGAT/ATCT	2	2	2	2	3

### Border Region Expansion and Contraction

**FIGURE 5:**
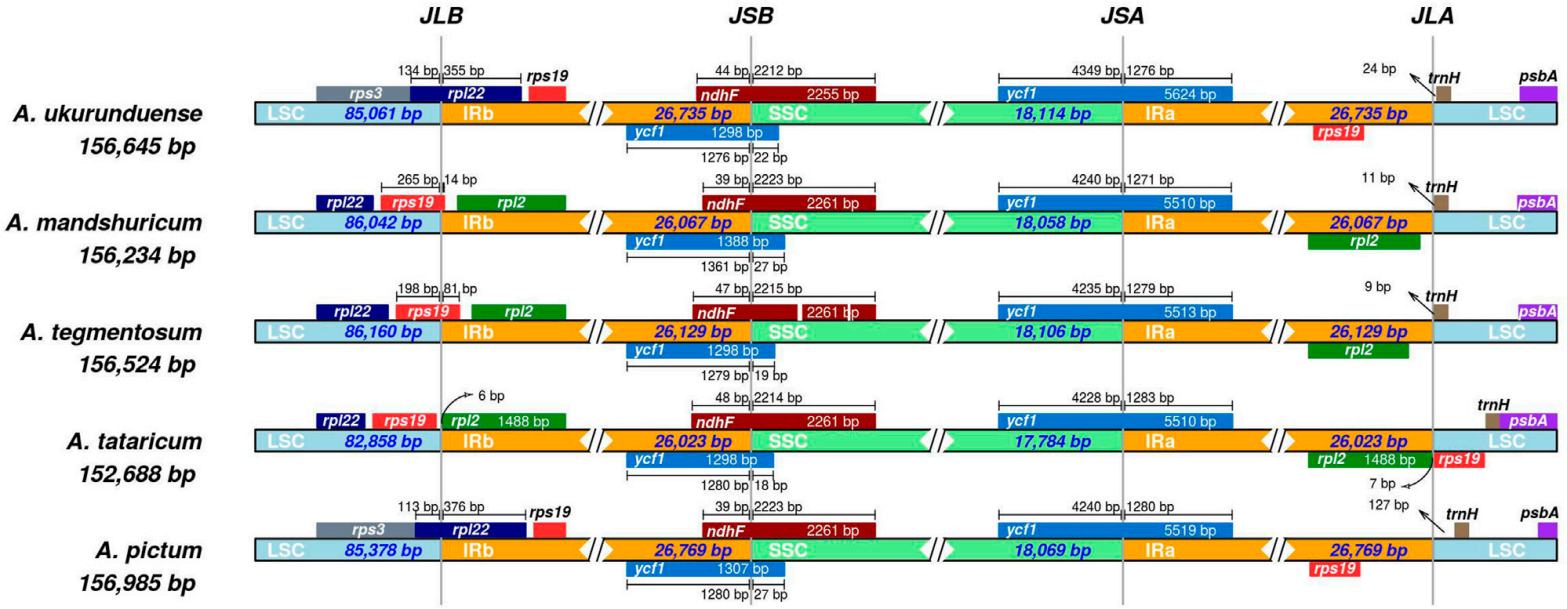
Comparison of the junctions of LSC, SSC, and IR regions among five cp genomes.

Differences in the size of the cp genome are mainly affected by the expansion and contraction of the border regions. In the current study, LSC/IRs and IRs/SSC borders and the genes located near junctions of different regions were compared and analyzed from the five *Acer* species cp genomes (Figure 5). The result indicated that the *rps19* gene was based on the junction of LSC/IRb in two of five species, while the *rpl22* gene was utterly situated at the junction of LSC/IRb in the two species of *A. ukurunduense* and *A. pictum*. The junction of IRb/SSC was located in the overlap region of the *ycf1* gene, and the gene was relatively conserved. The *ycf1* gene at the SSC/IRa junction and *trnH* gene at the right of the IRa/LSC junction in five species and *rpl2* gene in *A. ukurunduense*, *A. tataricum*, and *A. pictum* were compared. Deletion of the *rps19* gene occurred in *A. mandshuricum* and *A. tegmentosum*. The structure and gene content experienced no significant expansion or contraction of IR regions of *A. ukurunduense* and *A. pictum*, differing from the other three, which have similar gene boundary structures. These genomes presented obvious variances at the junctions, the gene sequence in LSC/IRb junction from 5 to 3′ is *rps3*, *rpl22*, and *rps19* in *A. ukurunduense* and *A. pictum*, while the other three species are *rpl22*, *rps19*, and *rpl2*. Furthermore, a comparison of the border regions was made among *Acer* species. During this analysis, the adjacent genes and some remarkable variations were discovered. The *rpl2* gene was absent from the regions of IRa and IRb in *A. ukurunduense* and *A. pictum*.

### Analysis of Nucleotide Diversity

**FIGURE 6:**
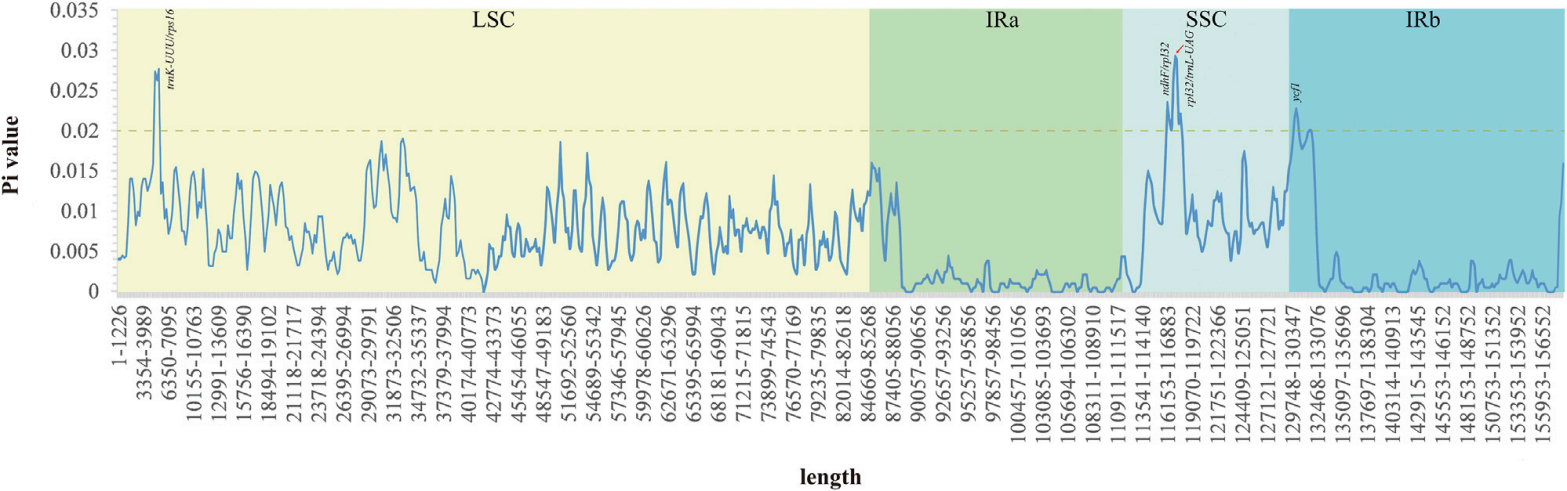
Nucleotide variability across the cp genomes of the five *Acer* species studied. The highest values were annotated based on the localization of predicted genes of the *A. ukurunduense* cp genome.

The Pi values ranged from 0 to 0.029 in the five *Acer* species (Figure 6 and Supplementary Table S6). This result showed that the species in the order *Acer* could undergo rapid nucleotide substitution. The IR regions had unobvious nucleotide variability compared with the SSC and LSC regions. There are four variable regions (*trnK-UUU/rps16*, *ndhF/rpl32*, *rpl32/trnL-UAG*, and *ycf1*) with Pi values exceeding 0.02, and all divergent regions reveal higher nucleotide diversity, which is observed in the intergenic region.

### Comparative Genome Analysis

**FIGURE 7:**
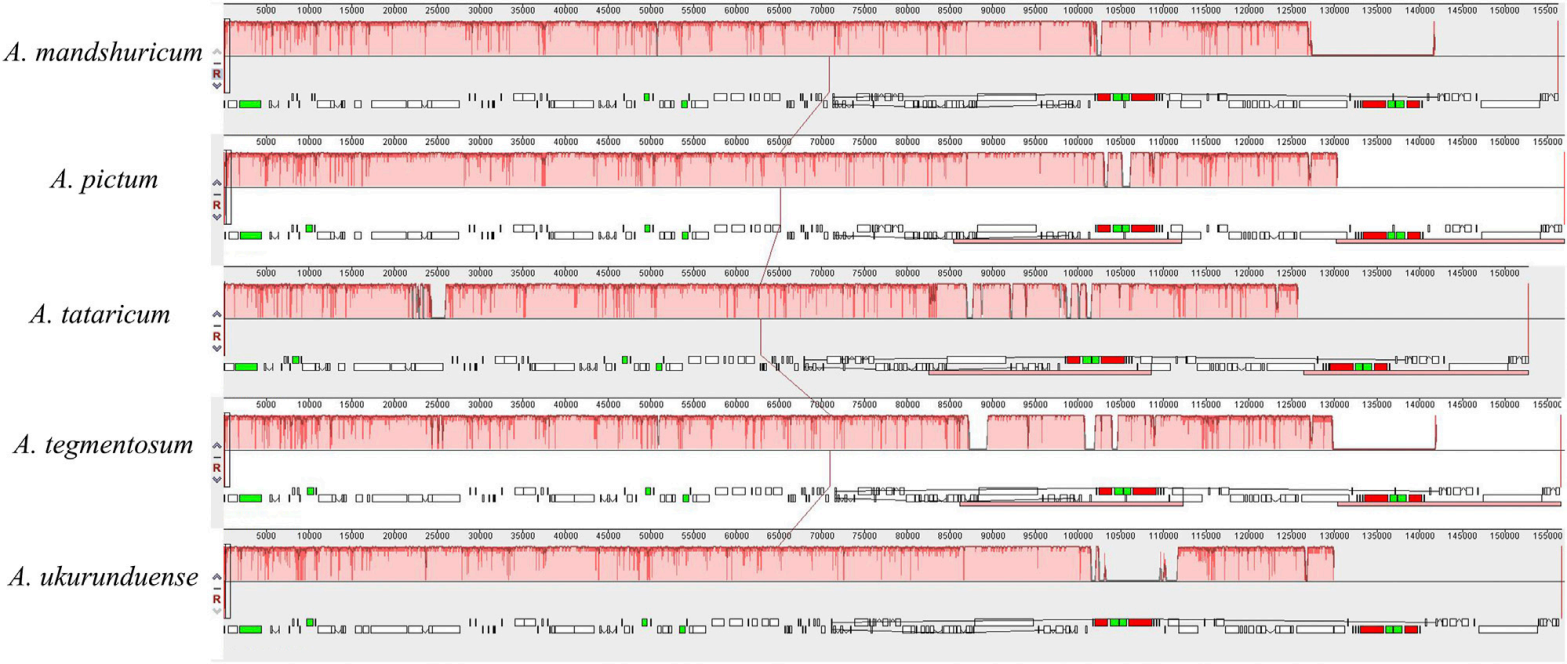
Comparison of the genome structure of five *Acer* species using the MAUVE program.

**FIGURE 8:**
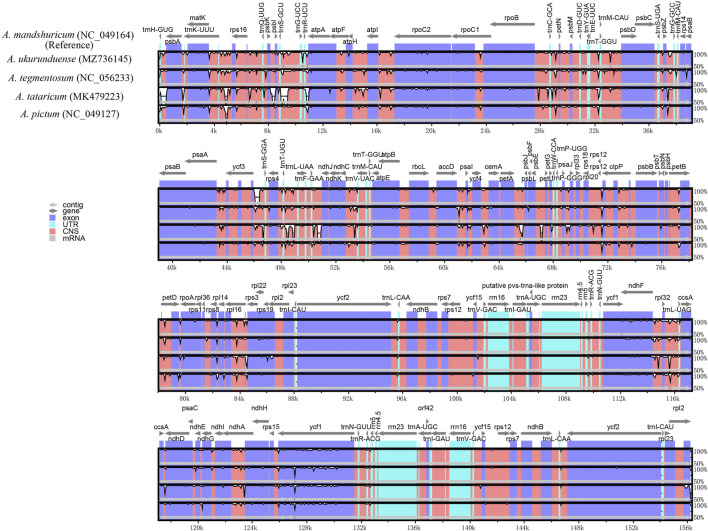
Visualized alignments of five cp genomes of *Acer* using mVISTA, with *A. mandshuricum* as the reference. The horizontal axis indicates the five species’ cp genome of the alignment. The vertical scale represents the percentage identity, ranging from 50 to 100%.

Comparative genomic analysis and the available DNA sequences make it possible to have a comprehensive view of the *Acer* genus. The cp genome sequences of *A. ukurunduense* were compared to *A. mandshuricum*, *A. tataricum*, *A. pictum*, and *A. tegmentosum* based on the MAUVE program (Figure 7). The results show that the structures of the cp genomes of the five *Acer* species are extremely similar and have good collinearity. By conducting the mVISTA program analysis (Figure 8), some of the high divergent regions were monitored, including *matk/rps16*, *trnQ-UUG/rps16*, *trnQ-UUG/psbI*, *trnS-GCU/atpA*, *atpH/atpI*, *psbZ/trnG-GCC*, *ycf3/trnS-GGA*, *trnT-UGU/trnL-UAA*, *trnF-GAA/ndhJ*, *petA/psbJ*, *ndhF/trnL-UAG*, and *ycf1.*

### Phylogenetic Analysis and Divergence Time Estimation

**FIGURE 9:**
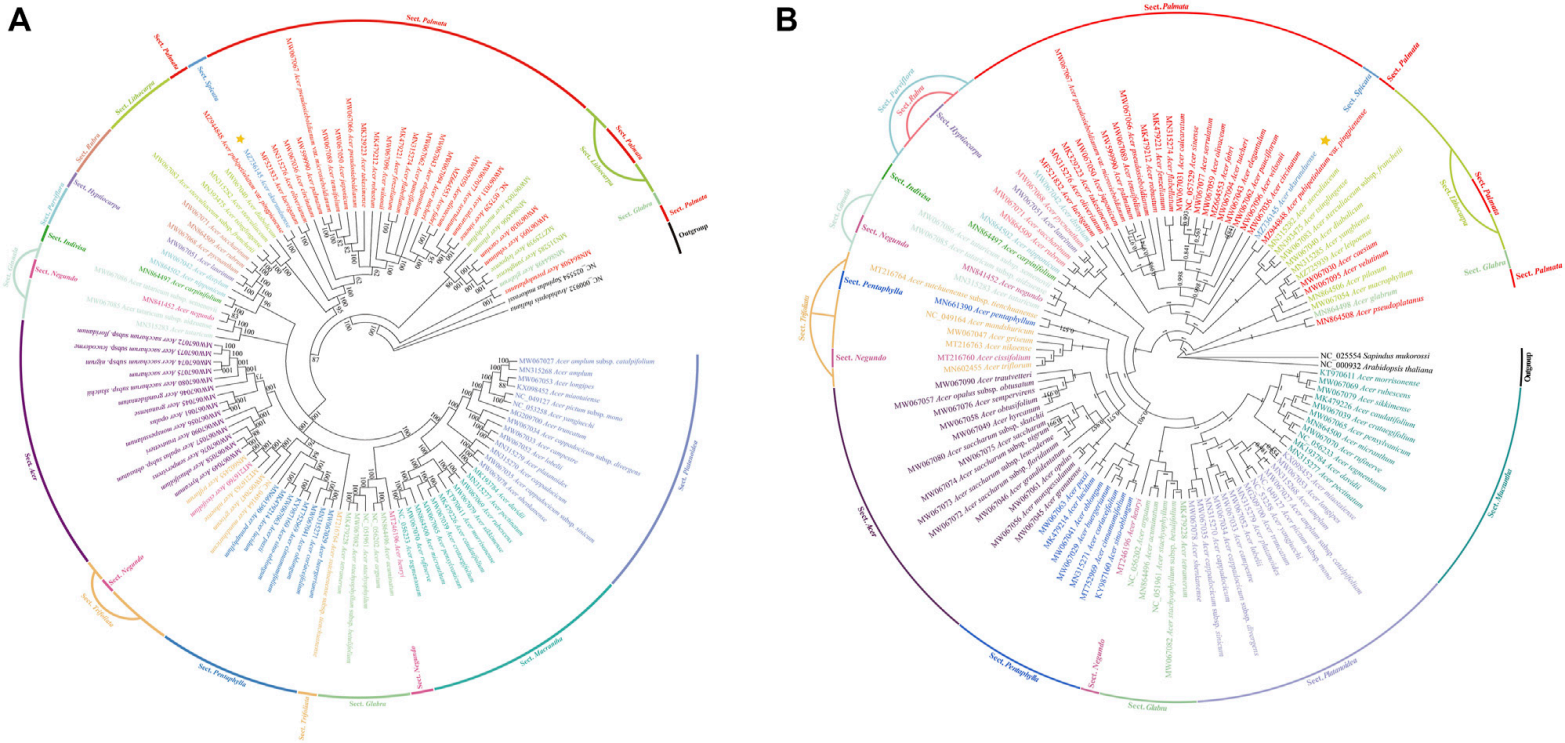
Phylogenetic trees based on 107 species of the whole-cp genome sequences using maximum likelihood (ML) **(A)** and Bayesian inference (BI) **(B)** analyses. Numbers at nodes are values for bootstrap support. Sequences from *Sapindus mukorossi* and *Arabidopsis thaliana* served as outgroups. The position of *A. ukurunduense* is indicated by a yellow star.

A total of 105 *Acer* species cp genomes and two species (*Sapindus mukorossi* and *Arabidopsis thaliana*) as outgroups were constructed from 15 Sect. using the ML tree and BI tree, respectively (Figures 9A,B). The two datasets (ML and BI) topologies generated similar structures. Fourteen species of Sect. *Platanoidea* were clustered together. Eleven species of Sect. *Macrantha* were clustered together. The ML and BI trees show that Sect. *Platanoidea* and Sect. *Macrantha* were able to gather into single bands. Sect. *Macrantha* is closely related to Sect. *Platanoidea*, followed by Sect. *Glabra*. Seven species of Sect. P*entaphylla* were clustered together. Five species of Sect. *Trifoliata* were not clustered into monophyly. Four species of Sect. *Trifoliata* were clustered together, and *A. sutchuenense* was separated into a single clade individually. Thirteen species of Sect. *Acer* were clustered together. The Sect. *Pentaphylla* is closely related to the Sect. *Trifoliata*, followed by the Sect. *Acer*. Sect. *Palmata* and Sect. *Lithocarpa* are closely related. As one of the only two species in Sect. *Spicata*, *A. ukurunduense* is more closely related to Sect. *Palmata*. The species of Sect. *Negundo* are closely related to Sect. *Glabra*, Sect. *Trifoliata,* and Sect. *Ginnala*, respectively. There are relatively close relationships among Scet. *Parviflora*, Sect. *Indivisa*, Sect. *Rubra*, and Sect. *Hyptiocarpa*.

The result of the divergence time estimation of 107 species based on chloroplast sequences is shown in Supplementary Figure S2. The age of the crown of the *Acer* genus was estimated to be in the upper Oligocene period, approximately 28.97 million years ago (Mya). The separation between Sect. *Platanoidea* and Sect. *Macrantha* occurred at 24.98 Mya. The divergence between Sect. *Glabra*, Sect. *Pentaphylla*, Sect. *Trifoliata,* and Sect. *Acer* was dated to 24.6 Mya. The estimated divergence time for Sect. *Palmata* with the most species was 25.66 Mya. *A. ukurunduense* and *A. pubipetiolatum* var. *pingpienense* diverged at 22.11 Mya.

### Molecular Markers Based on the Hypervariable Regions of Acer Cp Genomes

**FIGURE 10:**
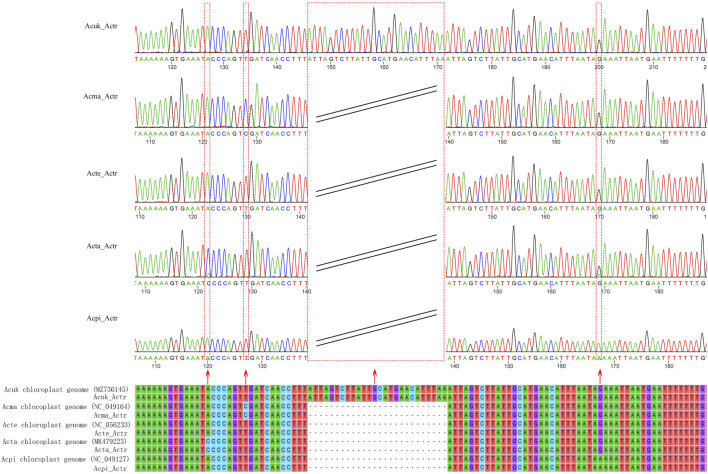
Alignment of the sequences of the PCR products amplified using the primer of *Acer*TS and the reference sequences. The red arrows indicate SNPs, Indel loci, and their corresponding peaks. Acuk: *A. ukurunduense*; Acma: *A. mandshuricum*; Acte: *A. tegmentosum*; Acta: *A. tataricum*; Acpi: *A. pictum*.

This study developed a specific molecular marker for accurate identification based on the hypervariable regions of the cp genomes of five *Acer* species from northeast China. Agarose gel electrophoresis detection showed that the size of the amplified fragment was the same as that of the target fragment (Supplementary Figure S3). Sequencing of PCR products showed six SNP and two insertion-deletion mutations (Indel) locus in the sequences of the five species. In addition, surprisingly, we discovered that three SNP loci and an Indel loci in the amplified sequence accurately identified the five species (*A. mandshuricum*, *A. tataricum*, *A. pictum*, *A. tegmentosum*, and *A. ukurunduense*) (Figure 10).

## Discussion

### Cp Genome Structure

There are some genes with unknown functions in this genome, such as *ycf1*, *ycf2*, *ycf4*, and *ycf15*. In most plants, the IRb and SSC boundary lie on the *ycf1* gene, which may be associated with the expansion of the IRb/SSC boundary (Kumar et al., 2018). In the cp genome of *A. ukurunduense*, we also observed the *ycf15* gene, which exists in most angiosperms but whose function remains unknown (Shi et al., 2013; Jung et al., 2021). Compared with the LSC and SSC regions, GC content in the IR region was the highest, mainly due to four rRNA genes occupying a greater area.

### Comparative Genomics

Codon usage bias is often used at unequal frequencies in all genomes and is well documented across species from all three domains of life (Duret, 2002; Chen et al., 2004). Selection operates on synonymous mutations because they influence various biological processes, some of which are linked to the expression of proteins and nucleic acid composition (Plotkin and Kudla, 2011; Zhou et al., 2016). In addition, amino acid hydrophilicity, tRNA abundance, gene position, gene expression rate, and base group composition are prime factors affecting codon usage. The choice of codon biases directly impacted nonuniform ribosome decoding rates on mRNA levels, which in turn influenced the protein construction that directly affects protein function in diverse biological processes. Hence, codon usage is a critical factor and, through translation, affects the mRNA secondary structure. Thus, codon usage is closely related to the efficiency of translation and posttranscriptional processes (Yi Liu et al., 2021). In the genetic code, except for tryptophan and methionine, which have only one codon, all other amino acids have more than one degenerate codon. Therefore, there is a certain bias in selecting degenerate codons in the coding process. When the RSCU value is greater than 1, the codon is preferred. This finding was unexpected and suggests that nearly all amino acid codons in *A. ukurunduense*, cp genomes have preferences. The main reason is the transcription process error prevented by amino acid activity. In the cp genome, the natural selection and evolution processes dominated a very significant determinant in influencing codon usage bias, which is that the use of codons also affects the structure and function of coding proteins. The results showed that the cp genomes of *A. ukurunduense* contain some codons that encode necessary amino acids.

SSRs are molecular markers as a major tool with high variation within the same species that are used in investigating genomic polymorphism and polymorphism studies. Different species have different types and content of motifs. The majority of SSRs in all species were A/T mononucleotides. The presence of SSRs in the cp genome is conducive to resolving genetic diversity between related genera and increasing the power of interspecific studies, possibly in combination with other informative nuclear genome SSRs (Lohne and Borsch 2005).

### Comparative Genomics

Almost all land plants have relatively conserved cp genomes. However, the arrangement of genes in the junctions of cp genomes differs from one species to another. A large IR expansion has also been reported in angiosperm lineages containing *Erodium* (Blazier et al., 2016), *Passiflora* (Rabah et al., 2019), and *Pelargonium* (Zhu et al., 2016). The IR regions will expand and contract, causing some genes distributed to be not fixed in the IR region or single copy region, generating the difference in genome size. The phenomenon contributes to resolving the process of genome evolution (Dugas et al., 2015). Our analyses reveal that the IR boundary expands in *A. ukurunduense* and *A. pictum* compared to the other three species. In *A. ukurunduense* and *A. pictum*, the *rpl2* gene disappears, while the *rps19* gene replicates, and the LSC/IRb boundary moves to the *rpl22* gene position. The results of the correlational analysis show that the expansion and contraction of the IR regions may be the determining factor that led to the size and gene locus changes of cp genomes.

There existed no significant rearrangements and no large fragment inversion among the compared cp genomes, and the sequence of genes was consistent and highly conserved. In general, the alignment sequence divergence across assemblies reveals that the cp genome in *Acer* is relatively conservative, especially in the gene coding regions when compared with its noncoding counterparts. By analyzing highly variable regions, ten highly divergent regions may be unique barcodes applied to species identification, also providing phylogenetic information. We also analyzed the disparity of cp genomes. This result showed that the intergenic regions had more variations than the gene coding regions, which is due to most fragments in IR regions having a relatively low Pi value. Some of these regions have been previously studied in other species’ cp genomes. The *trnK-UUU/rps16* has been successfully used to confirm the evolutionary relationship and complete Latin name changes of the *Sium* alliance within the Apiaceae tribe Oenantheae (Spalik et al., 2009). The phylogenetic analysis based on sequencing data from different molecular markers *ndhF/rpl32* and *rpl32/trnL-UAG* is also separate in the families Aristidoideae and Eragrostideae, and in the genuses *Fagopyrum* and *Dolomiaea*, respectively (Shen et al., 2020; Fan et al., 2021; Kuan Liu et al., 2021; Guo et al., 2022). The *ycf1* has been employed to assist in inferring the evolution of *Pinus*, *Hoya*, and *Curcuma* (Liang et al., 2020; Odago et al., 2021; Zeb et al., 2022). Those highly variable regions can be crucial molecular markers for closely related plants and contribute to generally phylogenetic relationship analysis research in the *Acer* genus.

### Phylogenetic Analysis and Estimated Divergence Time

Based on the development of next-generation sequencing technologies, cp genomes have been widely used to clearly explain plant phylogenetic relationships and evolution (Burke et al., 2016; Du et al., 2017). The phylogenetic tree was constructed based on the maximum likelihood (ML) approach with high bootstrap support values. The BI tree reveals the evolutionary position of *A. ukurunduense* and its relationship with other *Acer* species. ML and BI trees are broadly similar in the evolutionary classification of *Acer*. The results showed that *Acer, Macrantha,* and *Platanoidea* are monophyly, similar to previous studies (Figures 9A,B) (Gao et al., 2017; Areces-Berazain et al., 2021). The genetic relationships of most species are very stable, which helps to further study the classification of *Acer*. However, we found that different analysis methods can lead to changes in the relationship between Sect. The relationship between Sect. *Trifoliata* and Sect. *Pentaphylla* is closer to Sect. *Acer* in the ML evolutionary tree, whereas in the BI tree, the three Sects. are all related. The cut-off value for the condensed tree in the ML tree is 50%. If the parameter is increased to 60%, the relationship results will be consistent with those in the BI tree. Li et al. (2019) further inferred that Sect. *Trifoliata* may have evolved from Sect. *Pentaphylla* and should be incorporated into Sect. *Pentaphylla* is based on the bioinformatics analysis of 500 nuclear sites. However, Xia et al. (2022) did not support merging Sect. *Trifoliata* into Sect. *Pentaphyll*. Therefore, we suggest that further studies on the genetic relationship between Sect. *Trifoliata* and Sect. *Pentaphylla* can be performed based on nDNA or even whole gene data. In addition, the previous study indicated *A. ukurunduense* was closely related to Sect. *Palmata* based on plastomes and nuclear sequences for *Acer* species (Areces-Berazain et al., 2021). Our results strongly supported the conclusion. *Acer pictum* subsp. *mono* is traditionally considered a sister to *A*. *yangjuechi.* Yu et al. (2020) proposed *A*. *pictum* subsp. *mono* and *A*. *yangjuechi* as sister species according to the “local varieties”. Compared with previous research results, this study, for the first time, confirmed the phylogenetic relationship of *A. ukurunduense* in *Acer*, and the phylogenetic relationship of other species was consistent with the findings of Xia et al. (2022). The comparison between the ML and BI trees is sufficient to confirm the close phylogenetic relationships of the Sect. *Glabra*, Sect. *Platanoidea*, and Sect. *Macrantha*. Sect. *Acer*, Sect. *Pentaphylla*, and Sect. *Trifoliata* were also closely related, which is consistent with the results of recent studies (Areces-Berazain et al., 2020). The divergence time of the *Acer* genus basically coincides with the previous study by Renner et al. (2008). Furthermore, our result is in conformity with the previous study that found *Acer* diversified at a constant rate during the Oligocene and that diversification may not have been affected by the climatic cooling (Areces-Berazain et al., 2021). This phylogenetic data and divergence time could be useful for resolving the phylogenetic evolutionary relationships of Aceraceae and for the rapid and accurate classification of valuable germplasm resources.

### trnK-UUU/rps16 as a DNA Marker Used for Five *Acer* Species Identification

DNA molecular markers are short and relatively conserved fragment sequences in the plasmid sequence and distinguish species by stable mutation sites and insertion deletions. DNA molecular markers are widely used in traditional plant identification, accurate identification of medicinal plants, and invasive alien species. The hypervariable regions can be used as potential molecular genetic markers for identifying and evolving closely related species (Turktas, 2012; Park et al., 2017; Li et al., 2018; Chen et al., 2019). Through the previous analysis of ten sequences with highly variable regions, we preliminarily inferred that only one highly variable region (*trnK-UUU/rps16*) could be used to distinguish five species. This was proved by a sequencing comparison of *trnK-UUU/rps16* amplified fragments; the result fully verified the accuracy of the primers developed based on the *trnK-UUU/rps16* high variable region in identifying and providing a new method for discriminating *Acer* species. This method can also identify new and unique identification markers for other species and related species.

## Conclusion

In the present study, the cp genome of *A. ukurunduense* was investigated. By comparing and analyzing cp genome sequences of *A. ukurunduense* with those of other four species of *Acer* distributed in the forests of the Xiaoxing’an Mountains, China, our analyses reveal that *A. ukurunduense* was similar in gene size, content, and order, which has a typical quadripartite structure of most land angiosperm cp genomes. SSR analysis can exploit valuable information to develop a higher rate of mutation DNA markers for genetic diversity surveys, distinguish different germplasms, and conduct other ecological and evolutionary studies of *A. ukurunduense*. The contraction and expansion that occurred in the boundary regions indicated the size variation of the genome compared to the selected Aceraceae species. Moreover, phylogenetic evolution of the 105 cp genomes in *Acer* strongly supports the close relationship between different species. Additionally, experimental verification of the newly developed molecular marker shows that the *trnK-UUU/rps16* molecular marker can accurately identify five *Acer* species, which is of great significance to the taxonomic study of *Acer* species. These conclusions give valuable information for developing phylogenetic and genetic tools for further research on Aceraceae plants.

## Data Availability

The datasets presented in this study can be found in online repositories. The names of the repository/repositories and accession number(s) can be found in the article/Supplementary Material.

## Supplementary Material

Supplementary Figure S1 Technology roadmap.

Supplementary Figure S2 Divergence time analysis based on the chloroplast genome.

Supplementary Figure S3 Agarose gel electrophoresis of *trnK-UUU/rps16* fragments amplified in five species.

Supplementary Table S1 NCBI accession number of the sequence used in phylogenetic analysis.

Supplementary Table S2 Pair of primers for amplifying DNA barcodes.

Supplementary Table S3 Location and length of cp genes containing introns in *A. ukurunduense.*

Supplementary Table S4 Codon usage of five *Acer* species’ cp genomes.

Supplementary Table S5 Simple sequence repeats of five *Acer* species’ cp genomes.

Supplementary Table S6 Nucleotide diversity analysis data.
