# Supplementary figures and images for: Structural Characterization of the Acer ukurunduense Chloroplast Genome Relative to Related Species in the Acer Genus
Source: Front Genet. 2022 Jul 14;13:849182. doi: 10.3389/fgene.2022.849182 (PMC9329572; doi:10.3389/fgene.2022.849182)

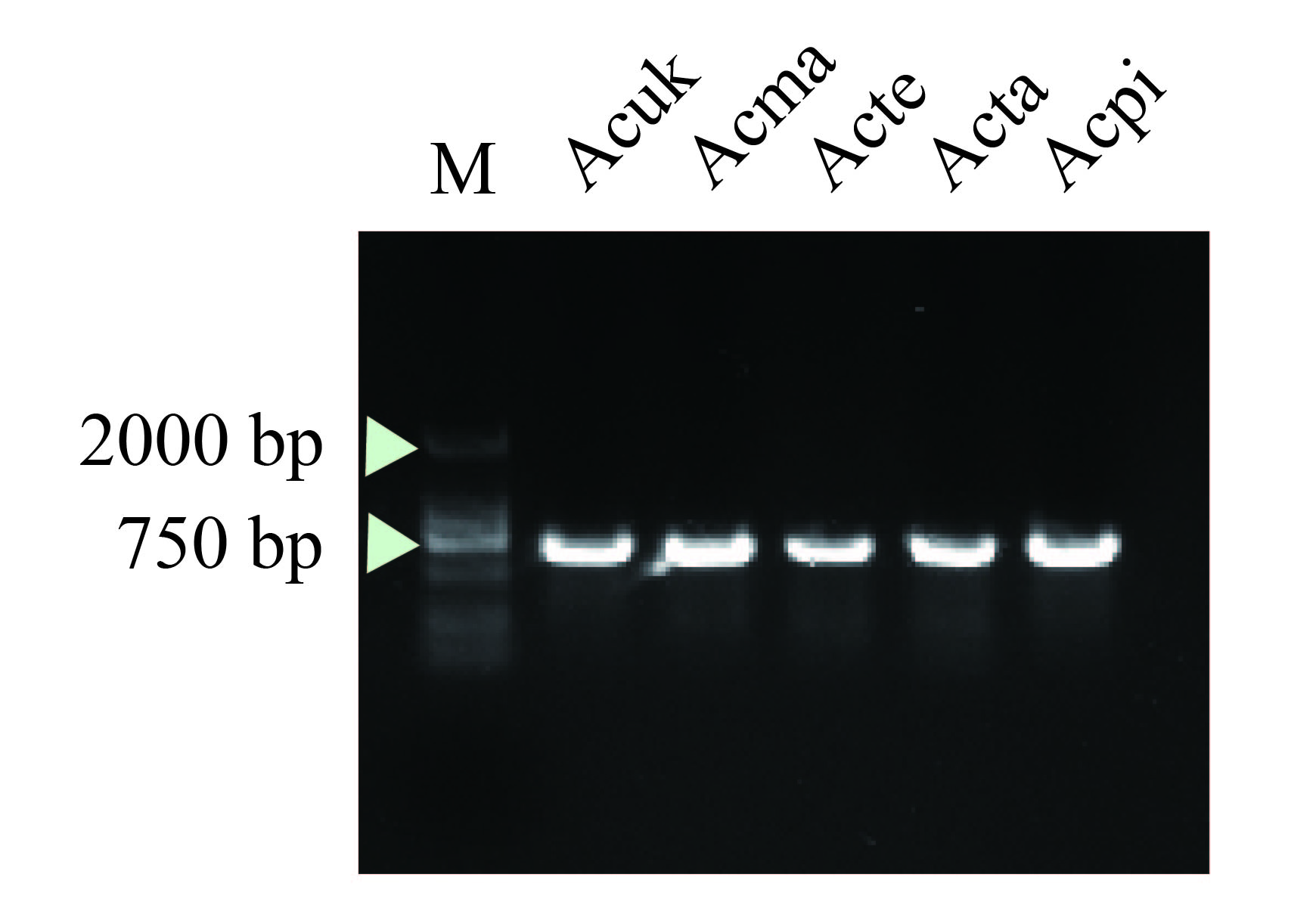

Supplement: Supplementary file 2 [file Image3.JPEG]

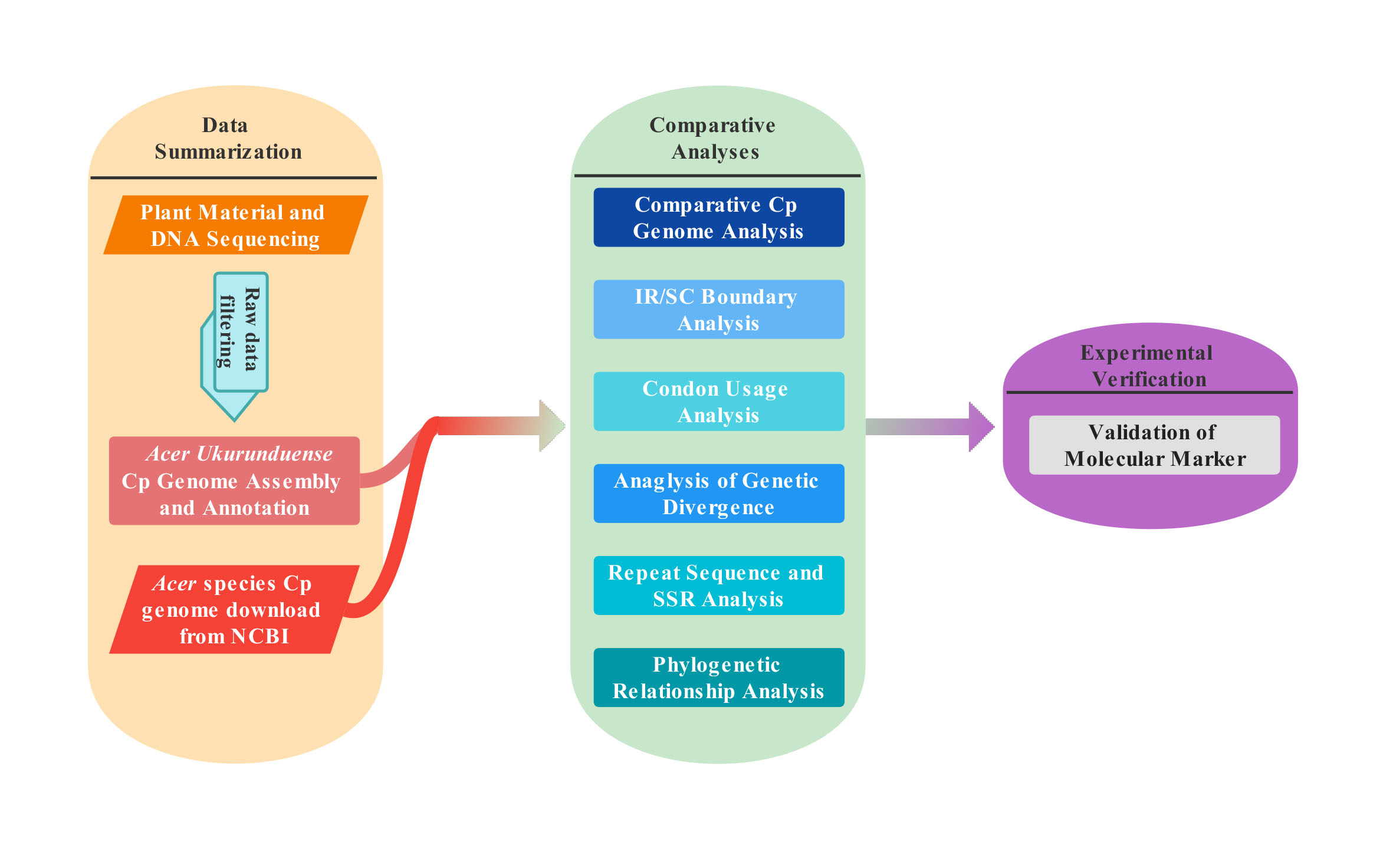

Supplement: Supplementary file 3 [file Image1.JPEG]

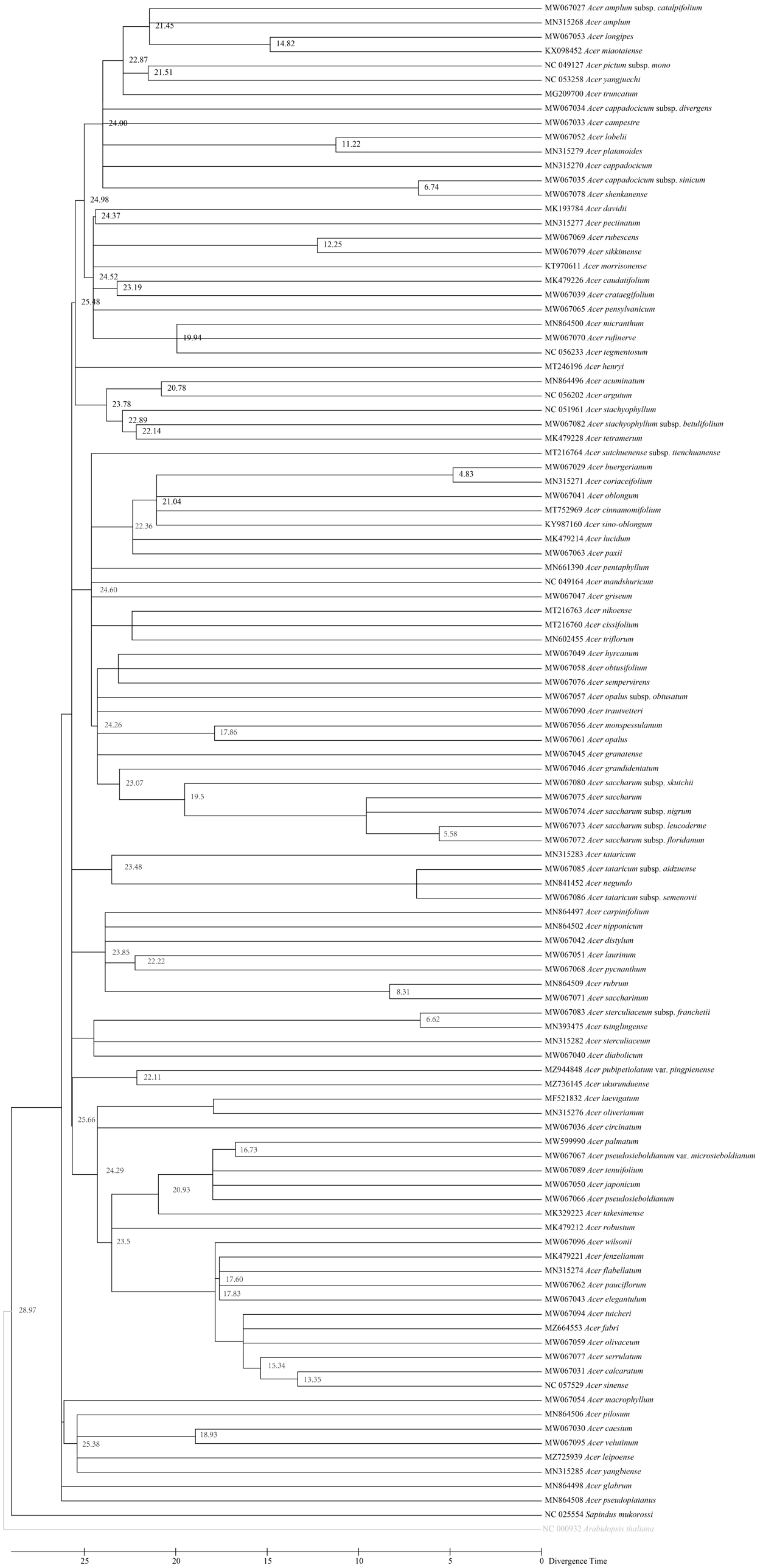

Supplement: Supplementary file 4 [file Image2.JPEG]
