# Supplementary material for: Structural Characterization of the Acer ukurunduense Chloroplast Genome Relative to Related Species in the Acer Genus
Source: Front Genet. 2022 Jul 14;13:849182. doi: 10.3389/fgene.2022.849182 (PMC9329572; doi:10.3389/fgene.2022.849182)
Supplement: Supplementary file 7 [file Table3.DOCX]

**Supplement Table 3.** location and length of cp genes containing introns in *A. ukurunduense*

| Gene | Start | End | ExonI | IntronI | ExonII | IntronII | ExonIII |
| --- | --- | --- | --- | --- | --- | --- | --- |
| *trnK-UUU* | 1832 | 4429 | 37 | 2526 | 35 |  |  |
| *rps16* | 5443 | 6500 | 39 | 809 | 210 |  |  |
| *trnG-UCC* | 9730 | 10511 | 24 | 710 | 48 |  |  |
| *atpF* | 12612 | 13939 | 161 | 761 | 406 |  |  |
| *rpoC1* | 21588 | 24390 | 430 | 742 | 1631 |  |  |
| *ycf3* | 44020 | 45987 | 126 | 726 | 231 | 732 | 153 |
| *trnL-UAA* | 48975 | 49573 | 37 | 513 | 49 |  |  |
| *trnV-UAC* | 53339 | 54002 | 39 | 588 | 37 |  |  |
| *clpP* | 71353 | 73422 | 71 | 878 | 291 | 604 | 226 |
| *petB* | 76324 | 77767 | 6 | 781 | 657 |  |  |
| *petD* | 77966 | 79198 | 9 | 699 | 525 |  |  |
| *rpl16* | 82680 | 84127 | 9 | 1037 | 402 |  |  |
| *rpl2* | 85794 | 87282 | 397 | 661 | 431 |  |  |
| *ndhB* | 95986 | 98200 | 775 | 682 | 758 |  |  |
| *trnI-GAU* | 103757 | 104785 | 42 | 952 | 35 |  |  |
| *trnA-UGC* | 104850 | 105729 | 38 | 807 | 35 |  |  |
| *ndhA* | 121451 | 123635 | 553 | 1093 | 539 |  |  |
| *ycf1* | 125562 | 131186 | 1195 | 105 | 4325 |  |  |
| *trnA-UGC* | 135978 | 136857 | 38 | 807 | 35 |  |  |
| *trnI-GAU* | 136922 | 137950 | 42 | 952 | 35 |  |  |
| *ndhB* | 143507 | 145721 | 775 | 682 | 758 |  |  |
| *rpl2* | 154425 | 155913 | 397 | 661 | 431 |  |  |
